# Actions Seen through Babies’ Eyes: A Dissociation between Looking Time and Predictive Gaze

**DOI:** 10.3389/fpsyg.2012.00370

**Published:** 2012-09-27

**Authors:** Moritz M. Daum, Manja Attig, Ronald Gunawan, Wolfgang Prinz, Gustaf Gredebäck

**Affiliations:** ^1^Research Group Infant Cognition and Action, Max Planck Institute for Human Cognitive and Brain SciencesLeipzig, Germany; ^2^Developmental Psychology Unit, Department of Psychology, University of ZurichZurich, Switzerland; ^3^Department of Psychology, Uppsala UniversityUppsala, Sweden

**Keywords:** infancy, action perception, eye movements, looking time, predictive gaze, dorsal-ventral

## Abstract

In this study, we explored the relation of two different measures used to investigate infants’ expectations about goal-directed actions. In previous studies, expectations about action outcomes have been either measured after the action has been terminated, that is post-hoc (e.g., via looking time) or during the action is being performed, that is online (e.g., via predictive gaze). Here, we directly compared both types of measures. Experiment 1 demonstrated a dissociation between looking time and predictive gaze for 9-month-olds. Looking time reflected identity-related expectations whereas predictive gaze did not. If at all, predictive gaze reflected location-related expectations. Experiment 2, including a wider age range, showed that the two measures remain dissociated over the first 3 years of life. It is only after the third birthday that the dissociation turns into an association, with both measures then reflecting identity-related expectations. We discuss these findings in terms of an early dissociation between two mechanisms for action expectation. We speculate that while post-hoc measures primarily tap ventral mechanisms for processing identity-related information (at least at a younger age), online measures primarily tap dorsal mechanisms for processing location-related information.

## Introduction

A hallmark of social-cognitive development is the ability to understand others’ actions flexibly and quickly. Infants have been shown to do so at an early age. Furthermore, at this early age, infants interpret the various components that constitute actions, such as intentions and goals as well as movements and means involved in achieving goals (Wagner and Carey, 2005).

One way to assess infants’ action perception is to measure their expectations about a forthcoming action, together with their responses when these expectations are met or violated. Two approaches have been predominantly used to do this. In the *post-hoc* approach, expectations are measured via *looking time*, for example in response to an observed action that is completed (e.g., Woodward, 1998). In contrast, the *online* approach is to measure expectations in anticipation of forthcoming action, for example, through *predictive gaze* during the observation of an ongoing action (e.g., Falck-Ytter et al., 2006). In the present study, we combined these two approaches in order to investigate how measures assessing infants’ expectations post-hoc and online of are related to each other in development.

### The post-hoc approach

The measurement of looking times as an indicator of infants’ cognition has been one of the most powerful tools in infancy research through the past 30 years. In a typical paradigm, infants are first habituated to a standard event. During the phase of habituation, infants build a representation of this specific event that allows them to form expectations about future events of a similar structure. Once a habituation criterion has been reached, two test events are presented that are variations of the habituation event. In one of the test events, the previously built expectations are met, in the other event, these expectations are violated. Longer looking times to one of the two test events indicate that the infants differentiated between the two events, that is, they could make use of the representation build previously and apply it to a novel situation.

In the context of infants’ perception of goal-directed actions, post-hoc measures were used to assess whether infants’ expectations about the outcome of an action are violated (resulting in longer looking time) or not when presented with test trials in which specific aspects of an observed action are altered compared to previously presented familiarization trials. To exemplify, Woodward (1998) habituated 6-month-olds to a hand reaching for one of two objects. In test trials, object locations were swapped and the hand either reached for the old object in a new location or the new object in the old location. Infants looked longer when the hand had reached for the new, relative to the old object, suggesting that they encoded the goal of the reaching action during familiarization and reacted with extended looking time when the agent changed its goal during test. Further studies using looking time have demonstrated that 6- to 12-month-olds encode goals of incomplete actions (Daum et al., 2008), the rationality of observed actions (Gergely et al., 1995), recognize the goal-directedness of successful, and failed reaching actions (Brandone and Wellman, 2009), and recognize goals of action sequences (Sommerville and Woodward, 2005), to list only a few. Looking time measurements allow a direct comparison between different sources of information (e.g., goal location vs. identity); however, infants’ responses are measured with low spatial and temporal resolution making it difficult to relate looking time data to underlying processes, a fact that has been discussed by a large set of research in the past already (e.g., Aslin, 2007).

### The online approach

The use of online prediction to investigate infants’ cognitive processes and sensorimotor integration has a similarly long tradition in infancy research. Studies using predictive reaching have shown that at the same age that infants start to reach for stationary objects, they start to reach for slowly moving objects (von Hofsten and Lindhagen, 1979). Furthermore, infants’ reaching movements have been shown to be predictive: Arm and hand movements are initiated before the target is within reaching distance, and are directed toward a future interception position (von Hofsten, 1980, 1983; Clifton et al., 1993). Infants’ reaching and grasping abilities have been shown to be predictive in various other aspects like adjusting the orientation (Lockman et al., 1984; von Hofsten and Fazel-Zandy, 1984; von Hofsten and Johansson, 2009) or the aperture size of the hand relative to a target (von Hofsten and Rönnqvist, 1988), and by predicting the weight of an observed object (Mounoud and Bower, 1974).

Measuring infants’ expectations online via predictive gaze is a relatively novel approach in infancy research (Gredebäck et al., 2010) although extensively used in adults (Flanagan and Johansson, 2003). This measure records an observer’s eye movements and measures the ability to predict ongoing events (e.g., looking at the final state of an event before accomplishment).

A growing number of eye tracking studies has reported infants’ abilities to predict the reappearance of objects that were shortly occluded (for a methodological review see Gredebäck and von Hofsten, 2007). This research has shown that infants as young as 4-month-olds already predict the reappearance of shortly occluded objects (Johnson et al., 2003; Rosander and von Hofsten, 2004). At the age of 6 months, infants’ predictions are no longer constrained to linear motion paths but they now quickly adjust their expectations to new non-linear motion paths (Kochukhova and Gredebäck, 2007).

A second application of measuring predictive gaze has been reported from studies testing infants’ categorization skills (McMurray and Aslin, 2004; Kovacs and Mehler, 2009; Addyman and Mareschal, 2010; Albareda-Castellot et al., 2011). McMurray and Aslin (2004), for example, developed an occlusion based anticipatory eye movement (AEM) paradigm where infants were presented with a training session in which one of two objects disappeared behind a T-shaped occluder and reappeared in one of two locations, depending on the identity of the moving object. Their results showed that infants learned to categorize different stimuli along a variety of stimulus dimensions such as color, orientation, or shape.

Measuring predictive gaze is specifically interesting in the context of investigating infants’ perception of others’ actions, as an action *per se* includes anticipation (von Hofsten, 2004). There are a number of studies using eye tracking to measure infants’ expectations online via predictive gaze. These studies have, for example, demonstrated that 6-month-olds predict that food will be brought to the mouth (Kochukhova and Gredebäck, 2010) and that 12- to 14-month-olds predict the goal of manual object displacements (Falck-Ytter et al., 2006; Melzer et al., 2012) and reaching actions (Gredebäck et al., 2009; Kanakogi and Itakura, 2011; Cannon and Woodward, 2012). The measurement of eye movements in general and of predictive gaze shifts in specific allows a detailed mapping of the spatial and temporal dynamics of infants’ action perception. In the same line as online measures offer advantages as compared to post-hoc measures, such as the track behavior on a fine-grained time scale, it has its limitations, for example, by constrained processing time and information.

### Comparing the two approaches

Post-hoc and online measures have not always revealed similarities in onset and development of action expectations, Cannon and Woodward (2012), for example, report predictive gaze shifts toward the correct target at the age of 11 months, while Woodward (1998) reports differences in looking times already being present at 5–6 months (or even earlier, as reported by Luo, 2011). One reason for this difference might be that the bases on which these expectations are built differ with respect to available information and time constraints. When measured post-hoc, expectations about an action are compared to the outcome of an action *after* it has been completed. The information about the action is *complete*. In contrast, when expectations are measured online, the measurement takes place *prior to* the completion of an observed action. The information about the action available is thus *incomplete*.

Given these differences, little is known about how these two measures relate to each other; whether they, for example, tap similar or different underlying cognitive systems. Only few studies have simultaneously used two different measures to assess infants’ action expectations (Gredebäck and Melinder, 2010; Paulus et al., 2011b). Gredebäck and Melinder (2010) demonstrated that 6- and 12-month-olds’ responses were more experience-dependent and developed later when measured online (via predictive gaze) than when measured post-hoc (via pupil dilations). Paulus et al. (2011b) showed that infants’ predictions did not reflect their looking times in an adapted version of the rational action paradigm as reported by Gergely et al. (1995).

This is first evidence that action perception abilities might be based on different underlying mechanisms and that more attention is required to map out what processes are tapped when investigating infants’ action perception.

Our aim here is to further explore the relation of the two approaches and the respective different measures that are used to investigate infants’ action perception. Looking time studies have shown that infants expect actions to be organized around goal identities rather than goal locations (Woodward, 1998). A great majority of predictive gaze studies within the domain of action perception, however, used single goals at fixed locations (Falck-Ytter et al., 2006; Cannon et al., 2012) or an assembly of similar goal objects at the same location (Gredebäck et al., 2009), leaving the question open whether predictive gaze is based on goal identity or location (see Paulus et al., 2011b; Cannon and Woodward, 2012, for exceptions). We adapted the looking time paradigm introduced by Woodward (1998) that includes two different goals at two distinct locations and combined it with a predictive gaze paradigm (similar to McMurray and Aslin, 2004; Kochukhova and Gredebäck, 2007).

## Experiment 1

In Experiment 1, infants’ action expectations were measured via looking times and predictive gaze shifts. The paradigm that was used primarily followed the logic of Woodward (1998); infants were familiarized with an agent moving toward one of two objects. In a subsequent test phase, the positions of the two objects were swapped and the agent either moved toward the old object on a new movement path (old goal/new path event) or to the new object on the old movement path (new goal/old path event). In order to be able to measure looking times and predictive gaze shifts at the same time, the original paradigm was modified as follows.

**Figure 1:**
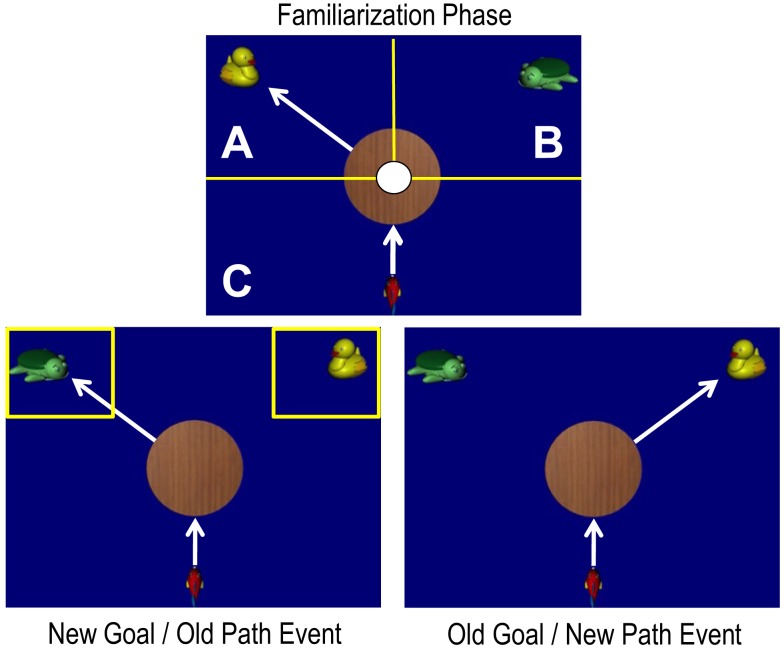
**An example of the stimulus presentation during familiarization trials (upper panel) and during the test trials (lower panels) in Experiment 1**. The upper panel additionally indicates there appearance AOIs (not including the white area covered by the inner circle) for the calculation of predictive gaze. Reappearance AOI A indicates the area of a goal-related prediction. Reappearance AOI B indicates the area of a non-goal-related prediction. Additionally, start area C is the area where the gaze originated from previous to prediction. In the lower left panel, the target AOIs that were used to measure the looking time to the respective targets are depicted additionally.

First, to trigger predictive gaze shifts, we followed the rationale of the occlusion based AEM paradigm (McMurray and Aslin, 2004) by adding an circular occluder in the center of the screen (similar to Kochukhova and Gredebäck, 2007). The agent moved toward the occluder, disappeared below the occluder and reappeared at the side of one of two targets, see Figure 1.

Second, in order to have a well-defined agent that completely disappears behind the occluder, we replaced the human hand by an animated agent, a small red fish, who moved fish-like (i.e., by wiggling its tail). Animated agents have been successfully used in studies investigating infants’ action expectations. Beginning with 6 months, infants are sensitive for the goal-directed behavior and the rationality of a wide range of human as well as animated agents (Csibra et al., 2003; Kamewari et al., 2005; Wagner and Carey, 2005; Csibra, 2008; Schlottmann and Ray, 2010). Using a paradigm similar to Woodward (1998), infants at the age of 6 months (Luo and Baillargeon, 2005) and even as young as 3 months (Luo, 2011) attribute goals to animated non-human agents. The agent that was used in the present study was designed to entail a variety of cues that have been shown to be help infants to perceive actions as goal-directed (e.g., self-propelledness, Biro and Leslie, 2007).

Third, we used a partially infant-controlled familiarization procedure and presented a fixed number of eight familiarization trials to each infant. This familiarization procedure has been successfully used in previous studies investigating infants’ goal attribution abilities using modified versions of the original Woodward paradigm (Hofer et al., 2007, 2008).

We tested 9-month-old infants as infants at this age show a robust goal attribution effect and anticipate action goals (Hunnius and Bekkering, 2010). Our hypothesis about the looking time results was clear. Based on the previous findings mentioned above, we expected infants to look longer at new goal/old path events compared to old goal/new path events. The hypotheses concerning predictive gaze were less obvious. Based on previous results, two outcomes concerning the infants’ predictions are conceivable. First, if infants attribute goals to an agent based on the identity of the goal as expected by their looking time (and as reported by Woodward, 1998), infants’ predictions should likewise be related to the identity of the goal (Cannon and Woodward, 2012). Second, and in contrast, 6-month-olds rapidly learn location-related associations in occlusion based AEM paradigms (McMurray and Aslin, 2004; Kochukhova and Gredebäck, 2007). Based on these findings one might expect that infants’ predictions would be related to the location of the goal.

### Method

#### Participants

Participants were 9-month-olds (*n *= 24; 11 girls; *M *= 9 months; 5 days; 8.20–9.15). Nineteen additional infants were excluded due to fussiness (resulting in too few trials, *n* = 16) or experimenter errors (*n* = 3). Infants had to administer a sufficient number of trials for two dependent variables, looking times and predictive gaze shifts. The fact that only the very first test trial could be analyzed with respect to predictive gaze shifts (see also below) was one major cause for the high exclusion rate. Furthermore, the large number of infants excluded from analysis (44%) is not unusual for eye tracking studies (McMurray and Aslin, 2004) and does not reflect the average exclusion rate as this was much smaller in Experiment 2, see below. Infants were recruited from a database of parents who had agreed to participate in infant studies.

#### Apparatus

The laboratory was unfurnished except for the test equipment. The infants were seated in a car safety seat (Maxi Cosi Cabrio), which was placed in front of the eye tracker. The stimuli were presented, and gaze was measured using a Tobii 1750 near infrared eye tracker (Tobii AB, Stockholm, Sweden) with an infant add-on (precision: 1°, accuracy: 0.5°, sampling rate: 50 Hz). A nine-point infant calibration was used. During calibration, a blue and white sphere expanded and contracted (extended diameter = 3.3°) in synchrony with a sound. Viewing distance was approximately 60 cm, display size was 25° × 21°. For the measurement of the looking times, a camera was positioned above the monitor and recorded a close-up view of the infants, which was displayed on a control monitor. Looking times were measured online by two trained observers (to assess reliability).

#### Stimulus material

Stimulus material was generated using the software CINEMA 4D R10 and BodyPaint 3D (Maxon Computer GmbH, www.maxon.net). It consisted of an agent (a red colored fish with yellow tail, 2.9° × 1.0°), an occluder (wooden colored; radius = 6.7°), and two targets (a yellow duck, 3.4° × 2.9°) and a green turtle, 4.3° × 2.9°), all presented on a blue background, see Figure 1. The whole experiment consisted of eight familiarization trials, one intermediate trial where the positions of the targets were swapped (swap trials) and six test trials. The familiarization and the test trials consisted of the following sequence. First, the agent first jumped up and down three times accompanied by a sound to orient the infant to the screen (initial phase: 4000 ms). The agent then moved swimming-like with a wiggling tail toward the occluder and disappeared behind the occluder (pre-occlusion movement from movement onset until the agent completely disappeared behind the occluder: 2480 ms), the agent continued to move under the occluder (occlusion time: 920 ms), reappeared from behind the occluder and moved toward one of the targets upon reappearing (post-occlusion movement from the first frame of reappearance until the arrival at the goal: 3400 ms). Once at the goal object, the agent poked it three times while the goal object remained static (poking time: 2520 ms). The agent then remained motionless until the trial was terminated. Looking time measurement started when the agent touched the goal object until the infant had looked away for 2 or 60 s had elapsed, at which time the trial ended.

Prior to the test phase, infants were shown that the goal positions were swapped with no agent present. Subsequently, two different test events were presented three times each, in alternating order. In the *old goal/new path* event, the agent moved on a new path toward the old goal (i.e., constant goal identity, changed goal location). In the *new goal/old path* event, the agent moved on the old path toward a new goal (i.e., changed goal identity, constant goal location). Goal object, movement path, goal locations, and test event presented first were counterbalanced between subjects.

### Data analysis

#### Post-hoc measure – looking time

Analogous to Woodward (1998) and as described above, looking time toward the whole display was coded during all familiarization and test trials online from a control monitor by two trained observers who were unaware of the condition (inter-rater agreement was 83%).

#### Online measure – predictive gaze shifts

Two gaze measurements were calculated based on the previous study investigating predictive gaze shifts in an occlusion paradigm (Kochukhova and Gredebäck, 2007). For this, the area of the video presentation was divided into three further areas of interest (AOI; see Figure 1, upper panel). AOI A and B (reappearance AOIs) covered each 90° of the occluder edge. These areas extended both inside and outside the occluder, covering all but the final 2° near the occluder center and extending outwards to cover the entire amplitude of the agent’s motion.

Gaze shifts were first categorized to be predictive or reactive. Predictive gaze shifts included all trials in which infants shifted their gaze across the occluder to target area A or B (see Figure 1) *before* the agent had been visible for 200 ms after occlusion. Reactive gaze shifts included all trials in which infants shifted their gaze across the occluder to target area A or B *after* the agent had been visible for 200 ms. This criterion was based on the average reactive saccadic latency to moving targets in adults (Engel et al., 1999) and infants (Gredebäck et al., 2006) and has been used in previous studies (e.g., Kochukhova and Gredebäck, 2007). Two primary scores, prediction rate and accuracy rate were calculated separately for each infant based on percentage scores.

##### Prediction rate

The prediction rate reports how often infants predicted the reappearance of the agent relative to the total number of attended trials. It is important to note that this measurement focuses on the timing of infants’ gaze shift over the occluder and does not take into account at which location the infants predicted the agent to reappear.

##### Accuracy rate

Second, the accuracy rate reports where infants predicted the agent’s reappearance; the number of predictions directed toward the target AOIs during familiarization was divided by the total number of gaze shifts (predictions and reactions) across the occluder. During familiarization, the *goal-related accuracy rate* (proportion of predictions toward the goal object) and the *non-goal-related accuracy rate* (proportion of predictions toward the other, non-goal object) were calculated. During test trials the *identity-related accuracy rate* (predictions being directed based on the identity of the goal during familiarization, i.e., toward the old goal on the new path) and the *location-related accuracy rate* (predictions being directed based on the location of the goal during familiarization, i.e., toward the new goal on the old path) were calculated.

#### Additional measures – specific looking times

Finally, the measurement of eye movement data allowed for a more detailed analysis of the position where infants were looking at what point in time during stimulus presentation. Accordingly, we were specifically interested in the proportion of looking time the infants spent looking at the areas of each of the two targets during the measurement of looking time. As for the looking times to the overall display, the measurement of these looking times was conducted after the agent had arrived at the respective goal during familiarization and test phase. In contrast to the measurement of the looking times to the overall display, this looking time measurement was calculated from the eye tracking data.

Additionally, to check whether the infants had seen the targets at their new locations, during the swap trial the looking time to the AOI around the two targets (target AOIs) was measured by counting the data points of infants gaze pattern that were located within this AOIs. The respective target AOIs are depicted in Figure 1 (lower left panel) and covered 7.2° × 6.4° starting from the upper and left/right border of the stimulus display.

#### Inclusion criteria

With respect to looking time, all familiarization and test trials were analyzed. With respect to the calculation of prediction rate and accuracy rate, all familiarization trials and the first test trial were analyzed. Because the agent reappeared in all of the test trials, infants received feedback about the agent’s behavior during the test trials immediately after the agent reappeared in the first test trial. For this reason, only the first test trial could be analyzed. In this first test trial, the infants had not yet received any feedback about the agent’s behavior after the positions of the targets had been swapped.

To be included in the data analysis, infants had to provide valid data for at least four out of eight familiarization trials, the swap trial, and four out of six test trials including the first test trial. For the looking times, these inclusion criteria had to be met during the phase when the looking time was measured. For the gaze shifts, a trial was classified to be valid if infants had tracked the agent prior to the occlusion passage and if they fixated one of the two possible target AOIs before or after the agent reappeared.

### Results

In the results section we first report how many children and trials were included in the data analysis. Then, the data of the looking time as our post-hoc measure followed by the predictive gaze shifts as our online measure and then directly compare the two measures. This is followed by a more detailed analysis of the looking times toward different AOI.

Looking times were analyzed using parametric analyses of variance (ANOVA) and *t*-tests. The analyses of the prediction and accuracy rates were performed using non-parametric Wilcoxon Signed Ranks tests. The data level did not justify parametrical analyses because the categorization of the gaze shifts to be predictive or reactive resulted in nominal scaled data.

#### Inclusion rates

All infants provided *looking times* during the first two familiarization trials (*Fam12*) and the last two familiarization trials (*Fam78*). During test, one infant provided data only for one out of all six old goal/new path trials, all other infants provided looking time data for all six test trials.

With respect to the analysis of *gaze shifts*, the following numbers of trials were included in the final analysis (percentage scores reflect number of included trials relative to number of presented trials): *Total number of trials* equaled 206 (95.4%). *Number of Fam12 trials* equaled 47 (97.9%); *Number of Fam78 trials* equaled 45 (93.4%); *Number of Test1 trials* equaled 24 (100.0%). During Fam12, one infant provided data from only one trial; during Fam78, one infant provided no data, and one data from only one trial; during Test1, all infants provided data from the first test trial. For the parametric analyses, the data of the infant who provided no data during Fam78 (1 out of 24 participants) was replaced by the respective grand mean to keep data loss minimal.

#### Post-hoc measure – looking time

Looking time decreased from Fam12 (*M *= 13.81 s, SD* *= 5.72 s) to Fam78 (*M *= 9.40s, SD* *= 3.62s), *t*(23) = 3.19; *p *< 0.01. A 2 × 2 [Test Event (new goal/old path; old goal/new path) × Order (new goal/old path events first; old goal/new path event first)] ANOVA revealed a significant main effect of test event, the infants looked longer at new goal (*M *= 9.57 s, SD* *= 3.97 s) than old goal events (*M *= 7.30 s, SD* *= 3.15 s), *F*(1, 22) = 6.77, *p* = 0.02. There was neither a main effect of order nor an interaction of order and test, both *F*s < 1. A non-parametric Sign test supported this result: 19 infants looked longer at new goal/old path events compared to five infants who looked longer at old goal/new path events, *p *< 0.01. As such, looking time results as a post-hoc measure replicate prior studies using the Woodward paradigm (Woodward, 1998; Luo and Baillargeon, 2005; Sommerville and Woodward, 2005) by demonstrating longer looking times when the agent moved along the same path as during familiarization to reach a new goal compared to when the agent moved on a new path to reach the same goal that was approached during familiarization.

#### Online measure – prediction rate

The infants performed predictive gaze shifts in roughly three-quarters of the trials, independent of the experimental phase. The prediction rate equaled 72.8% (SD = 22.2) over all familiarization trials and did neither change significantly from Fam12 (*M* = 72.9%, SD = 39.0) to Fam78 (*M* = 71.7%, SD = 35.6), *Z* = 0.0, *p* = 1, nor from Fam78 to Test1 (*M* = 62.5%, SD = 49.5), *Z* = −0.36, *p* = 0.72 (Wilcoxon test). These findings demonstrate that infants most often predicted that the fish will reappear from behind the occluder; this was true for both familiarization and test trials. In the following section we analyzed where the infants expected the agent to reappear.

#### Online measure – accuracy rate

Accuracy rates are presented in Table 1. During the familiarization, the goal-related accuracy rated was significantly higher than the non-goal-related accuracy rate during familiarization, *Z* = −2.15, *p* = 0.03. It did furthermore not change from Fam12 to Fam78, *Z* = −0.24, *p* = 0.98. The infants correctly predicted the agents’ movement during familiarization.

**Table 1:** **Mean accuracy rates in % (and Standard Deviations) during the first and the last two familiarization trials (Fam 12, Fam78) and the test trials (first test trial in experiment 1 and first two test trials in experiment 2**.

Experimental phase	Accuracy rate	Age						
		Experiment 1	Experiment 2					
		9 Months	All ages	9 Months	12 Months	24 Months	36 Months	Adults
Fam 12	Goal-related	47.9 (8.8)	40.3 (8.0)	47.8 (8.8)	39.6 (8.0)	29.2 (6.7)	54.2 (7.9)	30.4 (7.8)
	Non-goal-related	25.0 (6.7)	31.8(7.2)	30.4 (6.6)	37.5 (6.9)	27.1 (6.0)	31.3(7.9)	32.6(8.8)
Fam78	Goal-related	47.8 (8.2)	53.9 (8.4)	47.9 (9.3)	52.1 (8.2)	50.0(8.0)	62.2 (8.2)	54.2(8.5)
	Non-goal-related	23.9 (6.6)	18.9(6.3)	25.0 (7.4)	16.7(5.8)	12.5 (5.4)	19.6 (6.6)	20.8 (6.7)
Test	Identity-related	25.0 (9.0)	42.2 (8.5)	25.0 (7.4)	27.1 (8.0)	41.7(8.9)	58.3 (7.8)	58.7(8.3)
	Location-related	37.5(10.1)	34.3 (8.5)	47.9 (8.8)	41.7(9.2)	39.6 (9.0)	27.1 (7.4)	15.2(5.6)

As this analysis has shown that the infants had learned to correctly predict the reappearance of the agent during familiarization, the most relevant further analysis is to compare the (high) goal-related accuracy rate at the end of the familiarization phase (Fam78) to both the identity-related and the location-related accuracy rate in Test1. Comparing accuracy rates in the transition between familiarization (Fam78) and test trials (Test1) demonstrates that the goal-related accuracy rate was marginally higher than the identity-related accuracy rate in Test1, *Z* = −1.58, *p* = 0.11, and did not differ from the location-related accuracy rate in Test1, *Z* = −0.56, *p* = 0.58. These results indicate that infants performed less identity-related predictions in Test1 than goal-related predictions in Fam78, while the location-related predictions in Test1 did not differ from non-goal-related predictions in Fam78.

#### Comparison of looking time and eye movements

To directly compare looking time and predictive gaze, we compared the number of infants who looked longer at the new goal/old path events, thus did not expect a *change of identity* of the goal of the agent when measured post-hoc (19 out of 24, 79.2%) to the number of infants showing *identity-related predictions* in Test1 (6 out of 24, 25%) using a Chi-square test, χ^2^(1, *N *= 48) = 14.1, *p *< 0.001. The number of infants who performed identity-related predictions during Test1 was much smaller than the number of infants who performed identity-related looking times. Interestingly, all six infants who performed identity-related predictions the first test trial showed respective identity-related looking times and looked longer in the new goal/old path test events.

Although this result has to be interpreted with great care, as the number of infants per cell is very small, it indicates that those infants, who show identity-related processing of the agent when measured online, do so as well when measured post-hoc. In contrast, the reverse is not true, infants who show identity-related processing when measured post-hoc, do not necessarily show identity-related processing when measured online.

#### Additional measures – proportion of looking times toward different AOIs

Finally, to look more closely at the infants looking during the different experimental phases, we calculated the proportion of time infants spent looking toward the two target AOIs (looking time toward respective AOI divided by the total looking time as respectively measured by the eye tracker). During familiarization, infants looked longer at the goal object (proportion of looking time: *M* = 44.5%, SD = 13.9) than at the non-goal object (*M* = 15.2%, SD = 6.0), *t*(23) = 8.71, *p *< 0.001. This looking behavior did not change from Fam12 (goal object: *M* = 45.2%, SD = 18.2, non-goal object: *M* = 19.6%, SD = 9.2) to Fam78 (goal object: *M* = 41.9%, SD = 16.7, non-goal object: *M* = 11.5%, SD = 6.9) indicated by a main effect of target (goal object vs. non-goal object), *F*(1, 23) = 58.73, *p *< 0.001 and no interaction with phase (Fam12 vs. Fam78), *F *< 1. A main effect of phase, *F*(1, 23) = 11.59, *p* = 0.002, indicates that the proportion of looking toward the two objects decreased from Fam12 to Fam78. This result indicates that during the familiarization phase, the infants primarily looked at the goal object where the agent was.

During the swap trial the infants looked equally long at the goal object (*M* = 24.2%, SD = 14.4) and the non-goal object (*M* = 31.7%, SD = 16.8), *t*(23) = 1.31, *p* = 0.20.

Finally, the looking proportions during the test events were analyzed using a 2 × 2 × 2 [Test Event (old goal/new path vs. new goal/old path) × Target (old goal vs. new goal) × Order (old goal/new path event presented first vs. new goal/old path event presented first)] repeated measures ANOVA with test event and target as within-subjects factors and order as between factor. This analysis only yielded a significant Test Event × Target interaction, *F*(1, 22) = 51.59, *p* < 0.001. During old goal test events, the infants looked longer at the old goal (*M* = 38.8%, SD = 18.9) than at the other (new) goal (*M* = 10.6%, SD = 7.0). During the new goal test events, the infants’ looking behavior was reversed; they looked longer at the new goal (*M* = 36.3%, SD = 17.8) than at the other (old) goal (*M* = 12.9%, SD = 9.1). As during the familiarization trials, in the test trials, the infants primarily looked at the goal object where the agent was located. Furthermore, the infants looked at both objects during the swap trial and importantly, they looked equally long at the two targets during this trial. This indicates that the infants had observed that the positions of the two targets had been swapped.

### Discussion

The looking time results of Experiment 1 replicate previous findings (Woodward, 1998). When measured post-hoc, the infants looked longer when the agent moved on the old path toward a new goal compared to trials where the agent moved on a new path toward the old goal. Following the logic of Woodward (1998) infants built a representation about the agent’s goal during familiarization. During test, when the positions of the targets had been swapped, the infants’ expectation that the agent continues to move toward the old goal was met in the old goal/new path condition and was violated in the new goal/old path condition, resulting in extended looking times in the latter condition. In line with previous findings (Luo and Baillargeon, 2005; Csibra, 2008), this suggests that at the age of 9 months, infants are able to interpret a non-human agent’s behavior as goal-directed when expectations are measured post-hoc.

Interestingly, the infants’ predictive eye movements did not reflect their looking times. The results showed that the infants learned to correctly predict the reappearance of the agent during familiarization. However, during the first test trial infants showed a tendency to base their predictions on the location of the goal object as observed during familiarization.

A more detailed analysis of the looking times revealed that the infants predominantly looked toward the target that the agent was close to during both familiarization and test events. This finding has potential implications on the validity of the looking time task that will further be discussed in the Section “General Discussion.”

An important issue to be raised at this point is the fact that the present design does not allow for a distinction between goal-anticipations and path-anticipations. There are two reasons why we did not differentiate between these two measures. First, due to the restriction to only one test trial given by the paradigm, dividing the infants’ eye movements into goal- and path-directed gaze shifts resulted in a small number of test trials. Accordingly, the validity of such a measure would be limited. Second, more theoretically grounded, in a goal-directed action are goal and path mutually related. Anticipating the path an agent includes – at least in the present paradigm – the consideration of the goal the agent has. And vice versa, anticipating the goal includes the consideration of the path the agent takes. We did not differentiate between the two forms of anticipation.

To sum up, the main goal of the present Experiment1 was to compare an online with a post-hoc measure for infants’ action perception and to test whether infants base their predictions of the goal of an observed action based on the identity of the goal as suggested by previous findings using post hoc looking time measures (first hypothesis; e.g., Woodward, 1998), or on the location of the goal (second hypothesis; e.g., McMurray and Aslin, 2004; Addyman and Mareschal, 2010; Albareda-Castellot et al., 2011; Paulus et al., 2011b) as observed during familiarization. There is no definite answer to this question. The present results point toward a dissociation between looking time and predictive gaze and allow therefore a rejection of the first hypothesis. It is, however, less clear, what the basis of the infants’ prediction was as the results are ambiguous with respect to the two hypotheses. Furthermore, our conclusions are based on the performance of a few infants providing only one data point during the test trials. This might question the validity of the present data. For these reasons, we modified the paradigm used in Experiment 1 in order to replicated and extend the findings of Experiment 1 and to further explore the development of this potential dissociation.

## Experiment 2

In Experiment 2, we modified the paradigm from Experiment 1 to make it (a) a more prediction-oriented eye tracking paradigm and (b) to further strengthen processes of goal attribution by adding, for example, action effects. Additionally, we included a wider age range to investigate the developmental trajectory of responses when expectations are measured online.

### Method

#### Participants

We tested 9-month-olds (*n* = 24; 7 girls; *M* = 9.3; 8.17–9.13), 12-month-olds (*n *= 24; 9 girls; *M *= 12.5; 11.17–12.15), 24-month-olds (*n *= 24; 14 girls; *M *= 24.2; 23.15–24.14), 36-month-olds (*n *= 24; 8 girls; *M *= 36.8; 34.23–37.6), and adults (*n *= 24; 13 female; *M *= 24 years; 19–34 years). Additionally, fourteen 9-month-olds, six 12-month-olds, nine 24-month-olds, and two 36-month-olds were excluded from analysis due to fussiness or procedural errors.

#### Stimuli, apparatus, procedure, and data analysis

Stimulus material and procedure were adapted from Experiment 1 with the following modifications. We were concerned that the infants’ predictions in Experiment 1 were biased by the long inter-trial periods. These were caused by the measurements of the infants’ looking times resulting in periods up to 60 s depending on the infants’ looking behavior. In order to present trials in a higher frequency we shortened the trials and did no longer measure infants’ looking time.

Another reason why the infants did not predict the reappearance of the agent based on goal identity might have been that the stimulus presentation did not trigger goal attribution processes strong enough. For this reason, we strengthened these goal attribution processes by applying the following modifications: First, the targets were more distinct. In Experiment 1, both targets were animals; the infants might, thus, have processed both targets in terms of one category (animal) instead of two distinct targets (duck and turtle). We now followed more closely the targets as used by Woodward (1998) and replaced the turtle by an inanimate ball. Second, the agent’s poking of the goal object now caused a salient effect (during the poking, the goal object moved up and down while making a laughing sound). Previous research has shown that adding and effect to an unfamiliar action helps 6-month-olds to interpret the respective action as object-directed (Hofer et al., 2007; Jovanovic et al., 2007).

Finally, in order to be able to analyze more test trials, no more feedback was provided during the test events; the agent never reappeared from behind the occluder. This allowed us to repeat the test trial, thereby gaining additional data that will reduce noise and provide a more solid assessment of individual infants’ and children’s prediction and accuracy rate.

Goal identity, movement path, and goal locations were counterbalanced between participants. Accuracy and prediction rates were calculated as in Experiment 1. In addition, movement times were the same as in Experiment 1, the duration of the swap trial was 15 s, the test trials were presented for 10 s after the agent disappeared behind the occluder. Participants of all age groups were only told to watch the movies closely, without further instructions.

### Results

In the results section we first report how many children and trials were included in the data analysis, then, prediction and accuracy rates are reported. Finally, the individual age groups are analyzed separately. As in Experiment 1, the data level did not justify parametrical testing, accordingly, the non-parametric analyses were performed using Kruskal–Wallis tests and Wilcoxon Signed Ranks tests.

#### Inclusion rates

The following numbers of trials were included in the final data analysis. The *total number of trials* (maximum: 240 trials; including 192 familiarization and 48 test trials) equaled 229 (95.4%) for the 9-month-olds, 223 (92.9%) for the 12-month-olds, 227 (94.6%) for the 24-month-olds, 228 (95.0%) for the 36-month-olds, and 232 (96.7%) for the adults. *Number of Fam12 trials* (maximum: 48 trials) equaled 46 (95.8%) for the 9-month-olds, the 12-month-olds, the 24-month-olds, and the adults, each, and equaled 47 (97.9%) for the 36-month-olds. One 9-month-old, and one adult provided no data during Fam12, two 12-month-olds, two 24-month-olds, and one 36-month-old provided only one trial, all other participants provided data in both trials. *Number of Fam78 trials* (maximum: 48 trials) equaled 42 (87.5%) for the 9-month-olds, 44 (91.7%) for the 12-month-olds, 46 (95.8%) for the 24-month-olds, 42 (87.5%) for the 36-month-olds, and 47 (97.9%) for the adults. One 36-month-old provided no data during, six 9-month-olds, four 12-month-olds, two 24-month-olds, four 36-month-olds, and one adult provided only one trial, all other participants provided data for both trials. *Number of Test12 trials* (maximum: 48 trials) equaled 48 (100.0%) for the 9-month-olds, 42 (87.5%) for the 12-month-olds, 43 (89.6%) for the 24-month-olds, 46 (95.8%) for the 36-month-olds, and 44 (91.7%) for the adults. Six 12-month-olds, five 24-month-olds two 36-month-old, and four adults provided only one trial, all other participants provided data in both trials.

#### Online measure – prediction rate

Prediction rate equaled 74.7% (SD = 25.8) over all familiarization trials and age groups. Kruskal–Wallis tests revealed no differences between the age groups for the prediction rates over all familiarization trials [χ^2^(4, *N *= 120) = 7.07, *p* = 0.13], as well as for Fam12 [χ^2^(4, *N *= 120) = 9.03, *p* = 0.06], Fam78 [χ^2^(4, *N *= 120) = 4.16, *p* = 0.39], and Test12 [χ^2^(4, *N *= 120) = 2.58, *p* = 0.63]. Over all age groups, prediction rate did not change from Fam78 trials (*M* = 72.7%, SD = 39.5) to Test12 trials (*M* = 76.5%, SD = 36.1), *Z* = −1.02, *p* = 0.31 (Wilcoxon test). The prediction rate in Experiment 2 was comparable for the different age groups and for the different experimental phases. Similar to Experiment 1, the participants predicted in almost three-quarter of the trials that the fish will reappear from behind the occluder, both during familiarization and test trials.

#### Online measure – accuracy rate

##### Familiarization phase

**Figure 2:**
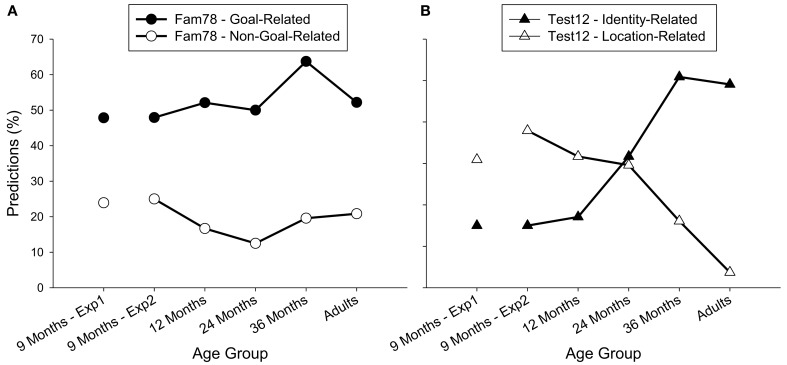
**Development of predictive gaze during the last two familiarization trials (A) and the test trials (B) across age groups in Experiment 1 and 2**. **(A)**: filled circles represent the goal-related accuracy rate; empty circles the non-goal-related accuracy rate. **(B)**: filled diamonds represent identity-related accuracy rate and empty diamonds the location-related accuracy rate.

We first checked whether the accuracy rates during the familiarization phase changed over age using Kruskal–Wallis tests. This was not the case, neither for the goal-related accuracy rate, χ^2^(4, *N *= 120) = 5.40, *p *= 0.25, nor for the non-goal-related accuracy rate, χ^2^(4, *N *= 120) = 2.65, *p *= 0.62, see Figure 2. Accordingly, to test whether the two accuracy rates differed from each other during familiarization, data was collapsed across age groups. As in Experiment 1, during familiarization, the goal-related accuracy rate was higher than the non-goal-related accuracy rate, *Z* = −5.66, *p *< 0.001 (Wilcoxon test).

##### Test phase

The same analyses were performed for the accuracy rates during the test phase. As can be seen in Figure 2, the identity-related accuracy rate increased over age, χ^2^(4, *N *= 120) = 15.37, *p *= 0.004, and marginally, the location-related accuracy rate decreased over age, χ^2^(4, *N *= 120) = 8.28, *p *= 0.08 (both Kruskal–Wallis tests). Accordingly, the accuracy rates of the different age groups were analyzed separately and are reported in further detail in the following section.

#### Analysis of individual age groups

As in Experiment 1, participants of all age groups had learned to correctly predict the reappearance of the agent during familiarization. In the next step, we compared the (high) goal-related accuracy rate at the end of the familiarization phase (Fam78) to both the identity-related and the location-related accuracy rate in Test12. The results indicate that the 9-month-olds showed a marginally significant change when the goal-related accuracy rate was compared to the identity-related accuracy rate, *Z* = −1.69, *p* = 0.09, but no significant change when it was compared to location-related accuracy rate: *Z* = −0.04, *p* = 0.97. The same pattern was found in the 12-month-olds, who significantly changed their looking behavior from Fam78 to Test12, indicated by a significant decrease of the identity-related accuracy rate, *Z* = −2.55, *p* = 0.01, but no change to the location-related accuracy rate, *Z* = −0.96, *p* = 0.37. In the 24-month-olds, looking behavior did neither concerning the identity-related accuracy rate, *Z* = 0.72, *p* = 0.42, nor the location-related accuracy rate, *Z* = −0.88, *p* = 0.38. In the 36-month-olds, no change of looking behavior was observed to the identity-related accuracy rate, *Z* = −0.59, *p* = 0.55, but here, a significant change to the location-related accuracy rate was found, *Z* = −2.85, *p* = 0.004. The same pattern was found in the adults where no change to the identity-related accuracy rate was observed, *Z* = −0.30, *p* = 0.77, but again a significant change to location-related accuracy rate, *Z* = −3.36, *p* = 0.001.

These changes from familiarization to test phase indicate, that the youngest two age groups continued to predict the reappearance of the agent on the basis of the previously observed location of the goal, while the oldest two age groups continued to predict the reappearance of the agent on the basis of the previously observed identity of the goal.

#### Looking times during swap trials

Additionally, we calculated the proportion of looking time toward the AOIs of the two targets and analyzed them by means of a 2 × 5 [Target (goal, non-goal during familiarization) × Age (9 months, 12 months, 24 months, 36 months, adults)] ANOVA that showed that the infants looked equally long at both objects (goal: *M* = 28.1, SD = 16.1; non-goal: *M* = 27.9, SD = 17.0), *F *< 1, and that the looking time decreased over age, *F*(4, 115) = 4.13, *p* = 0.004. This age effect is based on the shorter looking times of the adults (*M* = 20.4, SD = 12.8) compared to all other age groups (9 months: *M* = 30.3, SD = 17.6; 12 months: *M* = 28.5, SD = 17.4; 24 months: *M* = 31.7, SD = 17.6; 36 months: *M* = 29.2, SD = 15.0), indicated by LSD-corrected post-hoc tests, all *p*s < 0.01. No other differences between the age groups were significant. The interaction of the two factors was not significant. To ensure that the participants did look at the objects one sample *t*-tests against zero were performed for the looking proportion toward the two objects separately for each age group that were all significant, all *p*s < 0.001. All participants thus looked at the object and they looked equally long at both objects during the swap trials.

### Discussion

Experiment 2 showed that the 36-month-olds and adults predicted the reappearance of the agent in the test trials based on the identity of the goal of the observed action. In contrast, the 12-month-olds, and less clearly also the 9-month-olds based their predictions in the test trials on the location of the goal during the familiarization phase. The latter finding replicates the results of Experiment 1 indicating a dissociation between looking time as a post-hoc measure and predictive gaze as an online measure. Although infants do encode the identity of the action goal already at the age of 9 months when measured post-hoc, they base their predictions – though less clearly – on the location of the action goal. It is not before the age of 36 months, that children integrate goal identity in their predictions.

The ambiguous findings of the 9-month-olds might be explained by the fact that at this age, infants’ capacity to predict action goals is in a developing phase. Recent studies have shown that infants start to predict action goals at 6 months of age (Hunnius and Bekkering, 2010; Kochukhova and Gredebäck, 2010) and that this capacity continues to develop over the following months of life (Kenward, 2010; Paulus et al., 2011b), however reliable predictive gaze shifts are often not found before the age of 12 months (Falck-Ytter et al., 2006; Melzer et al., 2012) or even older (Gredebäck et al., 2009). In the present study, there was no difference in the overall rate of predictions between the two younger age groups, already the 9-month-olds showed predictions in more then 70% of the trials, so the 9-month-olds were principally able to predict the agents’ action. However, during the test phase, the 12-month-olds showed a clearer pattern of where their predictions were directed toward. The data finally shows that the 24-month-olds seem to be in a transition period, as their predictions were ambiguous. The looking times during the swap trials ensured that participants of all age groups have observed that the position of the targets has changed from the familiarization to the test phase. Potential causes and implications of this developmental trajectory from a dissociation between looking times and predictive gaze early in life to an association of the two measures later in life will be discussed below.

## General Discussion

In the present study, we compared post-hoc measures of children’s expectations about an observed goal-directed action with online measures concerning their predictions about the same action by combining a looking time paradigm with a predictive gaze paradigm. The looking time results from Experiment 1 replicated Woodward’s (1998) original findings. Nine-month-olds were shown to be sensitive to the identity of the goal of an observed action when measured post-hoc.

The results of the analysis of the infants’ eye movements contrast the looking time results and showed at the age of 12 months (and less reliably at the age of 9 months) infants predicted the reappearance of the agent based on the location of the goal during an observed action and that it was not until the age of 3, that this dissociation disappeared and that children predicted the reappearance of the agent after occlusion based on goal identity. These findings indicate that post-hoc measures and online measures used to investigate children’s action expectations are dissociated early in life. They further support the second hypothesis put forward in the introduction of Experiment 1 that early in life, infants continue to anticipate the reappearance of the agent in the test trials location-related.

This finding is, on the one hand, to some extent surprising as infants did encode the identity of the goal already at 9 months when their expectations were measured post-hoc, but they were not (yet) able to transfer this knowledge into their predictions. On the other hand, this findings is not that surprising as we know from previous findings that infants do take goal locations into account when predicting the future behavior of an agent (McMurray and Aslin, 2004; Addyman and Mareschal, 2010) and in the present paradigm, the infants were not only familiarized with the identity of the goal object but likewise with its location. The question remains, why this behavior changes with increasing age.

### From dissociation to integration

One answer to this question is that the computational processes that are involved in processing observed actions are dissociated early in life and become associated later: Early in life, action expectations measured online seem to be organized around goal locations whereas action expectations measured post-hoc around goal identities. With increasing age, children then generally organize their action expectations primarily around goal identities. The nature of this dissociation can either be interpreted as temporal or procedural.

A temporal interpretation implies that the dissociation between the two measures reflects two successive states on the processing timeline of one common underlying mechanism. This mechanism would act location-conservatively in an early processing phase during the observation of an action, and identity-conservatively in a later processing phase upon completion of the action. Action expectations measured post-hoc and online thus rely on a different amount of information available. During development, the sensitivity to action goals shifts backward on the processing timeline. Early in life, infants can derive goals only through post-hoc comparison of their expectations with an observation, with sufficient information and processing time available. Only later in life can they already derive goals more quickly online, during the observation of an ongoing action. This temporal interpretation is further supported by the findings that 6-month-olds, who were not yet able to anticipate the goal of a feeding action when measured online did differentiate between rational and non-rational feeding actions when measured post-hoc using pupil dilation where the processing time was less constraint (Gredebäck and Melinder, 2010). However, recent findings from a manual search task are not consistent with this temporal interpretation. When 2-year-old children were provided with additional time to process an observed event and to plan a response to search for a hidden object, performance did not improve (Mash et al., 2006).

In contrast, according to a procedural interpretation, the dissociation between the two measures reflects a dissociation between two different mechanisms involved, one for processing goal location and another for goal identity. These two mechanisms could be separate early in life and only later become integrated under the lead of the identity-related mechanism. This assumption is reminiscent of the notion of the two visual pathways (Mishkin and Ungerleider, 1982; Goodale and Milner, 1992) as a possible underlying mechanism. The *ventral* (*what*) pathway is associated with the processing of goal identity. The *dorsal* (*where*/*how*) pathway provides online spatial control of movements required for action execution and mediates the processing of goal locations. Both pathways are connected to the frontal eye field (Schall, 2002) that is involved in visual processing and inhibitory control (Schall et al., 2002; Muggleton et al., 2010). Evidence from animals (Schroeder et al., 1998; Chen et al., 2007) and humans (Rao et al., 2003) further suggests a dorsal-over-ventral advantage showing faster processing of location-related compared to identity-related information. These findings are mirrored by the reported developmental pattern that in the first year of life, visual processing seems to be driven primarily by the dorsal pathway (Leslie et al., 1998; Mareschal et al., 1999). Mareschal and Johnson (2003) further showed that young infants have difficulty in integrating information coming from the two streams. The authors suggest that it is the affordance of a target that determines which of the two representations is maintained. When the targets were non-manipulable objects like faces or asterisks, the infants primarily responded to changes in identity like color and not to changes in location. In contrast, when the targets presented were manipulable toys, infants primarily responded to changes in location and not in identity. In the present study we presented manipulable toys as targets. Accordingly, the young infants might have primarily responded to the location of the object, processed by the dorsal stream, when their expectations were measured online and could not integrate this with the information about the object identity, processed by the ventral stream. Mareschal et al. (1999) further suggest that a dissociation or a developmental lag only occurs when it is necessary to integrate two sources of potentially conflicting information, about location and identity. As they say “This explanation predicts that tasks requiring access to only one imprecise source of information or tasks that are performed with a visible object will not result in a developmental lag. In contrast, any task that calls for the integration of cortically separable representations will fail unless performed with a visible object or with precise cortical representations.” (p. 307). The advantage of the dorsal over the ventral stream found in the predictions of the younger children might therefore be based on the nature of the objects that were used as targets. The interpretation of Mareschal et al. (1999) are based on findings from the non-social domain and it remains a matter of further research to test whether the can be generalized to a social domain that includes animate cues as in the current paradigm and to test whether the dissociation found here can be modulated when non-manipulable objects are used as targets. It is, however, important to mention at this point, that in the present study, the objects used are the same for the two measures. Still, the infants do process the identity of the goal object when measured post-hoc but do only at a later age when measured online. The dissociation between the infants’ looking times and their predictions can, thus, not be traced back solely to the manipulability of the targets.

Both the temporal and the procedural interpretation are not necessarily mutually exclusive. The above-mentioned differentiation between the mechanism acting location- vs. identity-conservatively in the temporal interpretation of course entails a procedural element, as well as the as the procedural interpretation includes a temporal element, such as the differences in processing speed. The emphasis of the respective interpretations, however, lies on the processing mechanism in the procedural interpretation and on differences in the processing time in the temporal interpretation.

Within this context, it is finally important to emphasize the functionality of this early dissociation. When predicting action goals, the perception-action system has limited time to make accurate estimations of future events. Focusing on location (rather than identity) might be a useful “heuristic” often providing accurate and fast estimations. This does not mean that infants are ignorant of goal identity. With sufficient information and time, infants adjust their behavior according to the configuration of goal locations and identities.

### Looking times toward specific areas of interest

Another important aspect of the present study that needs to be discussed is the finding of Experiment 1 that the 9-month-olds primarily looked at the goal object where the agent was both during familiarization and test phase. The interpretation of this finding can take two major routes. First, a radical interpretation would imply that data from studies using looking time as a post-hoc measure for infants’ action expectations and their perception of goal-directed actions might be overly generous about infants’ knowledge. As mentioned earlier, looking time tasks measure infants’ expectations with fairly low spatial and temporal resolution (Aslin, 2007) and the results can easily be biased by low-level factors (see also Csibra, 2003). In the same line, it has been argued, that associative learning processes might subserve to a substantial part findings on early (social) cognition competences (Perner and Ruffman, 2005; Paulus et al., 2011a). For the following reasons, the present data might as well call into question the validity of the looking time tasks on action perception in general. During the familiarization phase, the infants spent more time looking at the familiarized (old) goal object than at the new goal object. This leads to an increase of the relative novelty of the new goal object in new goal/old location trials compared to old goal object in the old goal/new location trials and one cannot rule out that this relative novelty of surface features solely accounts for the increase in looking times in the respective test trials and that such a low-level explanation not only holds for the present data but for all data coming from studies using the same paradigm. However, there is evidence against such a low-level only explanation for the paradigm in general coming from previous looking time studies. These studies showed that at the same age, when infants do differentiate between the old goal/new path and new/goal/old path events when a familiar (grasping) action is performed by human agent, they do not show the same looking time pattern when a human agent performs an unfamiliar action (consisting in dropping the back of the hand on the object; Woodward, 1999; Hofer et al., 2005) or the human agent is replaced by an non-human agent performing the same action (e.g., mechanical claws, rods, occluders; Woodward, 1998). However, while the low-level factors of these studies can be assumed to be identical, the infants’ looking times were not.

We favor an alternative interpretation of our data suggesting a developmental trajectory with the identity-related action expectation measured post-hoc at an early age being a precursor of the identity-related action expectation measured online at a later age. This interpretation is supported by the finding that all 9-month-olds who performed identity-related predictions in Experiment 1 showed respective identity-related looking times (i.e., longer looking to changes in goal identity compared to changes in goal location). In contrast, the reversed pattern could not be found; infants who showed identity-related looking times did not necessarily perform identity-related predictions. Similar developmental trajectories including dissociations between post-hoc measures and online measures early in life have been reported in tasks testing infants’ knowledge about physical events. Expectations were measured online via manual search tasks where the children did not receive any feedback about the outcome of an observed event. Piaget, for example, has shown that infants do not manually search for hidden objects until they reach the age of 7.5–9 months. He concluded that it is not until this age that infants understand that hidden objects continue to exist (Piaget, 1952, 1954). In contrast, using post-hoc measures it has been shown that infants as young as 2.5 months do have some understanding about the continuity of hidden objects (Baillargeon et al., 1985; Wilcox et al., 1996). Similarly, when infants’ knowledge about physical solidity was assessed, infants differentiated between expected and unexpected events already at the age of 4 months when their knowledge was measured post-hoc (Spelke et al., 1992). In contrast, when tested in a manual search task, toddlers at the age of 2.5 years still failed when they have to predict the position of an object behind a barrier (Berthier et al., 2000). Further studies comparing toddlers’ knowledge about physical solidity in a within-subjects design showed that while toddlers failed to search at the correct location, they looked longer at an unexpected compared to an expected outcome of the same task (Hood et al., 2003; Mash et al., 2006). Keen (2003) concludes from these results that the perception of unexpected event outcomes seems to be a fundament upon which further knowledge about the world can be built. However, having knowledge (as assed via post-hoc measures) seems to be substantially different from being able to use that knowledge (as assessed via predictive gaze shifts or via manual search actions). Predictive gaze shifts, similar to manual search actions, not only require the evaluation of whether an observed event makes sense or not, the require an active – although not necessarily conscious – decision of where to shift gaze, a measure of the infants’ expectation before the outcome of an event is perceivable. This requires the consideration of multiple potential outcomes and the selection of the most appropriate one. Infants are able to infer the outcome of an uncompleted event, when their looking time is measured (Daum et al., 2008, 2009), or when they have to imitate previously observed incomplete actions (Meltzoff, 1995; Hamlin and Woodward, 2005). Accordingly, the conclusion by Keen (2003) fits as well for the present findings that the post-hoc evaluation of a task as measured via looking times builds the basis and is a prerequisite on which the online processing can be built upon.

The slope of these developmental trajectories might very well vary between different domains. Here we presented an animated object and found that looking time and predictive gaze were dissociated over several years. In their recent study, Cannon and Woodward (2012) report earlier identity-related predictions when infants were presented with a grasping hand instead of an animated agent. The results of this study showed that infants at the age of 11 months were able to predict the goal of the grasping hand based on its identity during familiarization. One might interpret this finding as evidence for an earlier understanding of human actions compared to non-human actions, the fact that children at this age correctly predict the goal of a grasping action might, however, also be caused by the fact that the infants were less constraint in the timing of their predictions. The infants did not have to take into account the precise timing of the grasping action once it stopped but could shift their gaze to one of the two objects after the hand stopped. In contrast, in the present study, both spatial and temporal aspects were needed to be integrated very precisely in order to correctly predict the agent’s behavior. This was not necessarily the case in the above-mentioned study. Furthermore, in the study by Cannon and Woodward (2012) the hand was never occluded during the presentation, while in the present study, in order to measure temporal and spatial aspects of prediction, the agent was occluded for a certain amount of time, what might have increased the difficulty of the task. Anyway, the prediction of action goals when measured online does still occur later, at the age of 11 months, than when measured post hoc (Woodward, 1998; Luo and Baillargeon, 2005; Luo, 2011). Further research will clarify how to what extent human and non-human actions are processed differently and on which basis predictions are made.

Finally, in the present study, we used a paradigm combining two visual measures of children’s action perception. The dissociation found is potentially not restricted to these two measures, quite the contrary, as previous studies have shown, other measures such as looking time and manual grasping (Hood et al., 2003; Mash et al., 2003) or, dilation and predictive gaze (Gredebäck and Melinder, 2010) show comparable findings. This suggests that it is likely that for almost all measures of infant behavior, dissociations will be found as long as they are based on different temporal constraints, different amounts of information and, accordingly, tap potentially different underlying processing mechanisms.

## Final Conclusion

In the present study, we explored the relation of two different measures used to investigate infants’ expectations about goal-directed actions. We compared post-hoc measures of infants’ expectations (via looking time) with online measures concerning their prediction (via predictive gaze). The looking times reflected identity-related expectations already at the age of 9 months. In contrast, predictive gaze pattern show that at a young age infants base their predictions primarily on the location of a goal object while it is only after the third birthday that predictive gaze reflects identity-related expectations as well.

## Conflict of Interest Statement

The authors declare that the research was conducted in the absence of any commercial or financial relationships that could be construed as a potential conflict of interest.
